# Release, Transfer, Fold: Using a Silicone Adhesive for On-Demand 3D Tissue Engineering

**DOI:** 10.1021/acsbiomaterials.5c01130

**Published:** 2025-10-11

**Authors:** Doris Roth, Benedetta Zampa, Romina Augustin, Daara Payandehjoo, Giancarlo Porcella, Ayşe Tuğçe Şahin, Anne M. van der Does, Janna C. Nawroth

**Affiliations:** † Helmholtz Pioneer Campus, Helmholtz Zentrum München, Neuherberg D-85764, Germany; ‡ Institute of Biological and Medical Imaging, Bioengineering Center, Helmholtz Zentrum München, Neuherberg D-85764, Germany; § Chair of Biological Imaging at the Central Institute for Translational Cancer Research (TranslaTUM), School of Medicine and Health, Technical University of Munich, Munich, Munich D-81675, Germany; ∥ Comprehensive Pneumology Center Munich, German Center for Lung Research (DZL), Munich D-81377, Germany; ⊥ PulmoScience Lab, Department of Pulmonology, Leiden University Medical Center, Leiden 2300 RC, The Netherlands; # Faculty of Engineering Sciences, University of Heidelberg, Heidelberg D-69120, Germany

**Keywords:** flexible tissue engineering, low-cost technology, primary cell cultures, adhesives, 3D, foldable

## Abstract

Conventional cell culture substrates are flat and rigid, locking cells in a permanent and unphysiological geometry. Advanced tissue culture models that emulate the dynamic and 3D environments of organs remain challenging to generate. Here, we establish flexible silicone adhesive films as versatile substrates that enable the on-demand release, transfer, and folding of cultured 2D tissues into 3D geometries. We rolled primary epithelial cultures into tubes, assembled cuboidal structures, and transferred primary endothelial cultures between culture environments for coculturing. Our approach provides an easy-to-implement platform for dynamic geometrical designs in tissue engineering.

## Graphical abstract

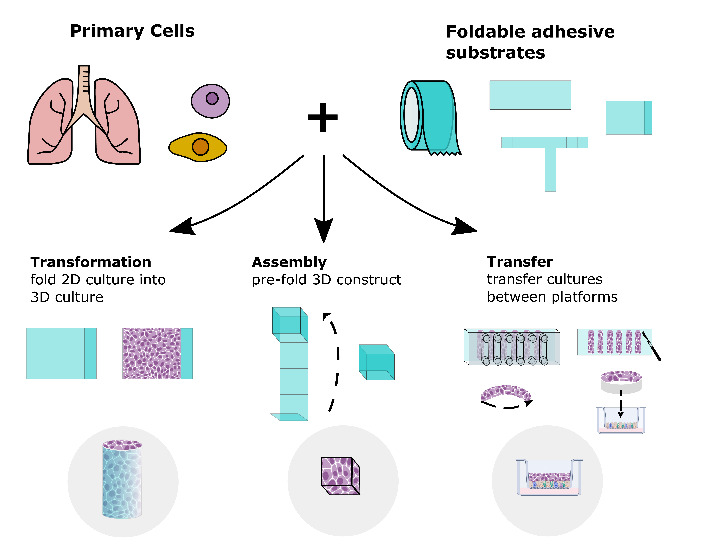

## Introduction

In the human body, organs and their tissues are three-dimensional (3D) and regularly deform through body movements, growth, and other dynamic processes. In contrast, materials used as substrates for *in vitro* human tissue models are prevalently two-dimensional (2D) and permanent in shape, such as cell culture dishes made from hard plastics. Advanced 3D culture systems, including microfluidic chips, bioreactors, and hydrogel cultures, better mimic the dynamic *in vivo* environment by allowing mechanical deformation, cellular remodeling, and self-assembly.^1,2^ However, these systems often require complex fabrication techniques, such as soft lithography and 3D bioprinting that may be challenging to implement in standard biological laboratories and workflows.^3^ Predesigned culture vessels can achieve physiologically relevant 3D environments, but, again, lock cells into a particular geometry and complicate fabrication, cell seeding, and maintenance.^4^ Finally, advanced coculture models face challenges in finding a common medium and mechanical cues that enable parallel maturation.^5^ Here, we address these challenges by leveraging a flexible 2D cell substrate that allows for the release, transfer, and 3D folding of mature tissues with minimal fabrication efforts. Our easy-to-implement approach uses silicone-based double-sided adhesive, commonly used to bond microfluidic devices^6^ and supporting the growth of immortalized cell lines,^7^ as a 2D substrate for primary human cell cultures that can later be transformed into new geometries. In three conceptual studies using lung epithelial and endothelial cells, we demonstrate cell culture on 2D sheets, the assembly of these sheets into 3D tubes and cubes, and their transfer into culture vessels containing other cell types to establish on-demand cocultures. In summary, our adhesive-based method offers a versatile, low-cost, and accessible platform for creating tissue models of variable geometries, with applications in both basic and potentially translational research.

## Materials and Methods

### Rapid Prototyping and Fabrication

We used clear pressure-sensitive silicone-based adhesive (SR-29) lining both sides of a polypropylene film (ARcare 94119, Adhesive Research) with a total thickness of 142 μm and an acrylic-based pressure-sensitive adhesive (AS-110) lining both sides of a polyester film (ARcare 90445Q, Adhesive Research) with a total thickness of 81 μm. All adhesive shapes were cut using a CAMM-1 Servo GX-25 cutting plotter equipped with a ZEC-U5032 (Roland) blade. Following cutting, the adhesive shapes were autoclaved and, after handling under sterile conditions, additionally sterilized with ethanol. Rectangles were prepared for 2D culture systems with the ability to roll into tubes. An additional cut was made in the top release liner, 1 mm from the edge, creating a defined culture area and a separate adhesive overlap area for tube formation. Cube nets were cut, and small incisions were made at each crease position to aid bending. For endothelial perfusion cultures, an adhesive rectangle was cut to match the dimensions of a bottomless six-channel slide (μ-Slide I/IV, ibidi). The adhesive was affixed to the autoclaved channel slide by using manual pressure.

### Cell Culture

#### Cells

Human primary small airway epithelial cells (hSAECs) and human primary pulmonary microvascular endothelial cells (hPMECs) were purchased from CellSystems (Lifeline FC-016) and Promocell (C-12281). Both companies ensure that their products meet the strictest European and international ethical standards, including obtaining informed consent from donors and protecting donor privacy.

#### Epithelial Cell Culture

PET membrane culture inserts (24-well; pore size of 0.4 μm, Transwell Corning) for control cultures and adhesive surfaces were coated overnight with 300 μg/mL human collagen type IV (Sigma-Aldrich). Expanded hSAECs were seeded at a density of 250,000 cells/cm^2^ and cultured in BEpiCM medium (ScienCell Research Laboratories). Once confluent, the medium was switched to PneumaCult-ALI medium (STEMCELL Technologies) supplemented with 10 μM DAPT (Thermo Fisher Scientific) for differentiation under submerged conditions, following established protocols.^8^ For air–liquid interface (ALI) control cultures in cell culture inserts (Transwell, Corning), the basal medium was replaced with a PneumaCult-ALI medium, and the apical side of the cultures was air-exposed to promote differentiation. In all cultures, the medium was replaced on Mondays, Wednesdays, and Fridays, an apical wash using PBS was performed on ALI cultures twice per week, and cells were differentiated for 17 days.

#### Endothelial Cell Culture

For endothelial cultures, purchased fully assembled channel slides with a tissue culture-treated polymer coverslip bottom (μ-Slide VI, ibidi) or fabricated channel slides (bottomless μ-Slide VI, ibidi) with an adhesive bottom were coated overnight with 50 μg/mL human fibronectin (Corning). hPMECs expanded in endothelial cell growth medium-MV2 (ECGM-MV2, PromoCell) were seeded into the channels at a density of 300,000 cells/cm^2^ and allowed to attach for 1 h. After attachment, cells were cultured under physiological low shear stress (2.3 dyn/cm^2^) for 5 days using the ibidi Pump System.

### 3D Scaffold Folding and Assembly

#### Tubes

After 17 days of differentiating the hSAECs on the rectangular shapes, cultures were rolled into a tube by removing the remaining release layer to create an adhesive overlap, aligning the cells at the borders.

#### Cubes

Cube nets were carefully bent along incisions to create creases for precise folding. Under sterile conditions, the release layers were removed, and the net was folded into a cube using tweezers before cell culture to not destroy tissue integrity upon folding the cube. The resulting cube was immediately immersed in coating solution and later seeded with epithelial cells as described in the Supporting Information and methods.

The resulting tube and cube cultures were stained for live imaging and later fixed in 4% paraformaldehyde.

### Culture Transfer

After the hPMECs were aligned on the double-sided adhesive sheet under flow, the adhesive was carefully peeled from the channel slides. Custom-shaped hPMEC sheets were then excised from the rectangular sheets by using a scalpel or dissection scissors. The remaining release liner on the opposite side of the cell layer was removed to expose the adhesive, allowing us to stick the culture to the side walls of insert cultures with tweezers, resulting in an endothelial ring perpendicular to the hSAECs and establishing a coculture. The resulting cocultures were fixed in 4% paraformaldehyde.

Additional details and method descriptions are provided in the Supporting Information.

## Results

We tested pressure-sensitive acrylic-based adhesive tape (PSAA) and silicone-based adhesive tape (PSSA), both commonly used in microfluidic cultures and known for their biocompatibility,^6^ and excised customized morphable culture surfaces using a cutting plotter (Figure 1A,B). Following autoclaving, the top release layer of the 2D sheets for tube cultures was removed, exposing the adhesive surface only where cell attachment is desired. The sheets were then glued into a cell culture dish by using a second adhesive to prevent floating (concept 1). For concept 2, the microfluidic chip was assembled by bonding a bottomless channel slide to the adhesive tape, and for concept 3 the cube was folded using tweezers (Figure 1C). We cultured donor-derived primary endothelial cells and epithelial cells on the exposed adhesive surface after coating it with extracellular matrix protein (Figure 1D,E). Primary human pulmonary microvascular endothelial cells (hPMECs) were aligned under shear flow in the microfluidic chip, and primary human small airway epithelial cells (hSAECs) were differentiated toward mucociliary pseudostratified epithelium in submerged static conditions (Figure 1E). Once ciliation was present in the epithelial cell cultures and endothelial cells aligned with the flow in the microfluidic chip, the engineered tissues were either transferred into coculture systems or folded into functional 3D tubes, demonstrating a versatile method for designing adaptable cell culture formats using donor-derived tissue (Figure 1F).

**Figure 1:**
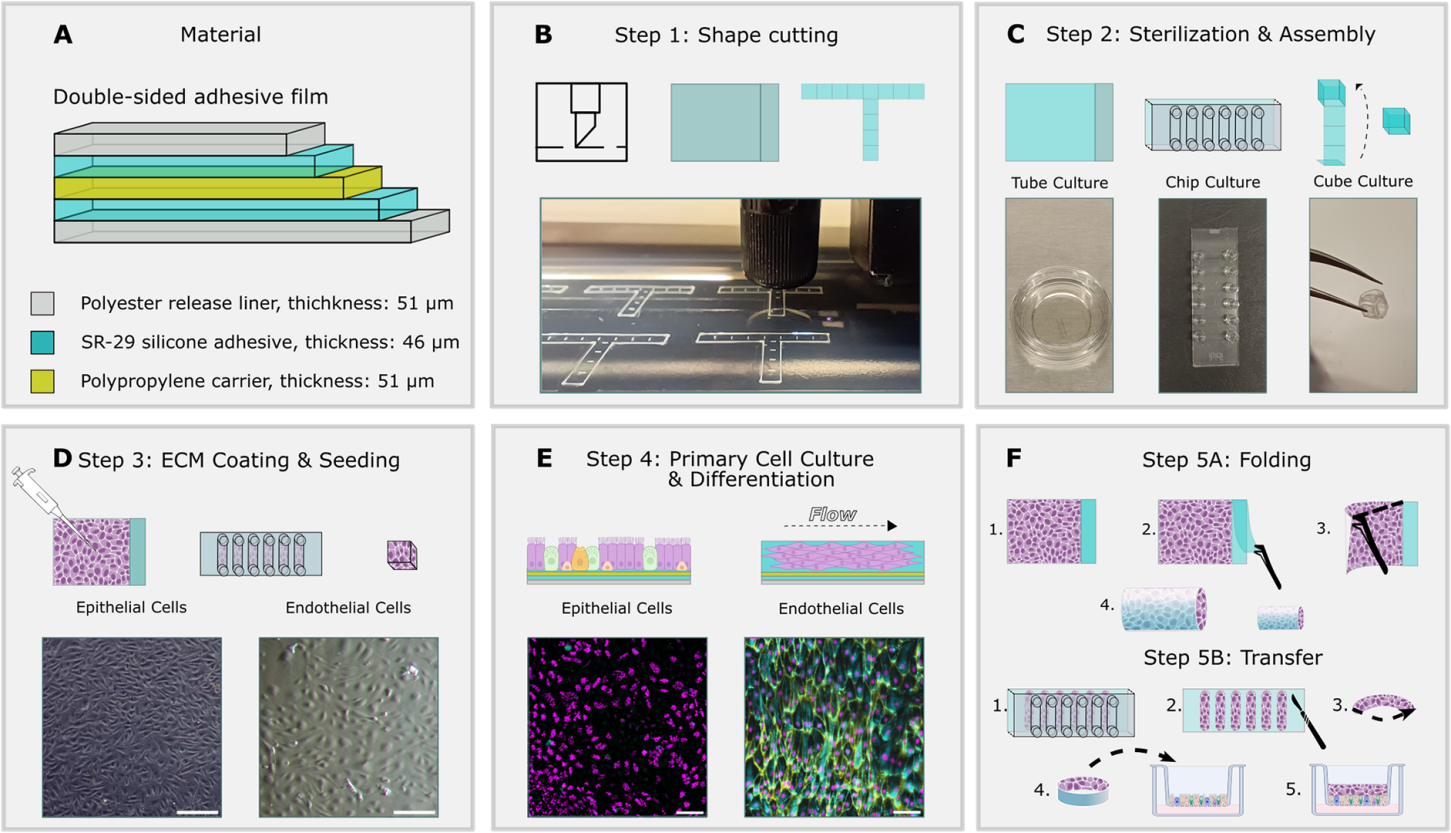
Schematic workflow of silicone adhesive cultures. (A) Composition of pressure sensitive SR29 silicone-based adhesive. (B) Step 1: Cutting of substrate shapes or release liner using cutting plotter; see methods for details. (C) Step 2: Following sterilization and disinfection, adhesive surfaces are exposed and (1) glued to culture dishes, (2) glued to microfluidic channels, or (3) assembled into 3D structures (3). (D) Step 3: Exposed adhesive surfaces are coated with extracellular matrix proteins and seeded. Brightfield images: primary lung epithelial and endothelial cells on PSSA 24 h post seeding. (E) Step 4: Cultures are differentiated (epithelial cells) or perfused with a medium (endothelial cells). Epifluorescence images: Left: epithelial cells after 17 days of differentiation on PSSA. Cilia (magenta; ATUB/α-tubulin), goblet cells (cyan; MUC5AC), and club cells (yellow; SCGB1A1). Right: endothelial cells on PSSA. Nuclei (magenta; DAPI), endothelial adherens junctions (yellow; VE-cadherin, aka CD144), F-actin cortex (cyan; phalloidin stain). (F) Step 5A: Upon ciliation, rectangular epithelial cell sheets are folded into tubes (concept 1). Step 5B: aligned endothelial cell cultures are released from the microfluidic chip by peeling the PSSA from the channel slide and are transferred to airway epithelial cell cultures. Scale bars: (D) 50 μm; (E) left image: 50 μm, right image: 100 μm.

hSAECs adhered successfully to both adhesive substrates and remained attached throughout the 17-day differentiation period under submerged conditions. However, only hSAECs cultured on the PSSA developed a differentiated mucociliary phenotype with multiciliated cells and secretory goblet and club cells, at levels comparable to submerged cells cultured in conventional culture inserts and approaching levels observed in standard, nonsubmerged, air–liquid interface (ALI) insert cultures^9^ (Figure 2A and SI, Figure S1). Functional analysis of ciliary activity demonstrated no significant differences in ciliary beating frequency between multiciliated cells cultured on PSSA and those cultured on conventional inserts (Figure 2B). Lactate dehydrogenase (LDH) assays revealed no significant adverse effects of the adhesives on cell viability (Figure 2C). Collectively, our findings indicate that PSSA, but not PSAA, is a suitable cell culture surface for the culture and differentiation of primary hSAECs.

**Figure 2:**
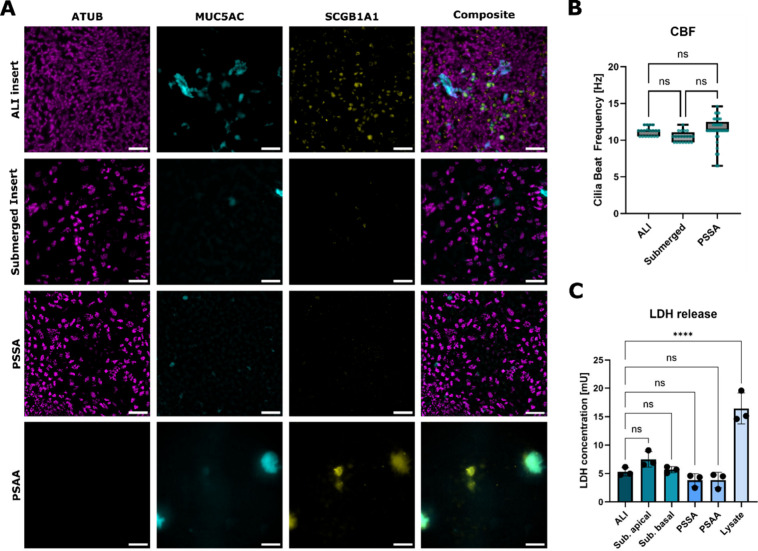
Differentiation of primary airway epithelial cells. (A) Representative immunofluorescence stainings of hSAECs at day 17 of differentiation in air–liquid interface (ALI), submerged on inserts, PSSA and PSAA. Cilia (magenta; ATUB/α-tubulin), goblet cells (cyan; MUC5AC), and club cells (yellow; SCGB1A1). (B) Quantification of average ciliary beat frequency. Data points represent pooled data from 3 to 5 culture replicates and a minimum of 5 FOVs per culture. Thick horizontal lines represent the median, bottom and top edges of the boxes represent the 25th and 75th percentiles, whiskers indicate the minimum and maximum. (C) Lactate dehydrogenase release in culture supernatants. The data points for each condition represent 3 independent culture replicates from 1 donor. The top of the column represents the mean. The whiskers represent the standard deviation. Significance was assessed using one-way ANOVA followed by Tukey’s multiple comparisons test; ****=*p* < 0.0001. Scale Bars: 50 μm.

Next, we tested whether PSSA can also sustain primary endothelial cells and the application of microfluidic perfusion. We cultured hPMECs on PSSA or, as a control, on standard cell-cultured treated plastic, in a microfluidic chip either statically or under perfusion at shear stress levels of 2.3 dyn/cm^2^ for 5 days. The shear stress levels were chosen to promote alignment of the hPMECs with the direction of the flow and formation of tight cell–cell junctions.^10^ After 5 days of constant perfusion, cells in perfused culture conditions were elongated and aligned with the direction of the imposed shear flow and exhibited well-developed adherens junctions (Figure 3A) whereas static control cultures exhibited less elongated cells with random orientations and less developed adherens junctions (Figure 3B). No significant adverse effect of the adhesive on the viability of the hPMECs was observed (Figure 3C). These results demonstrate that PSSA supports microfluidic applications, such as Organ-Chips, and enables the culture of aligned, viable endothelial cells under flow.

**Figure 3:**
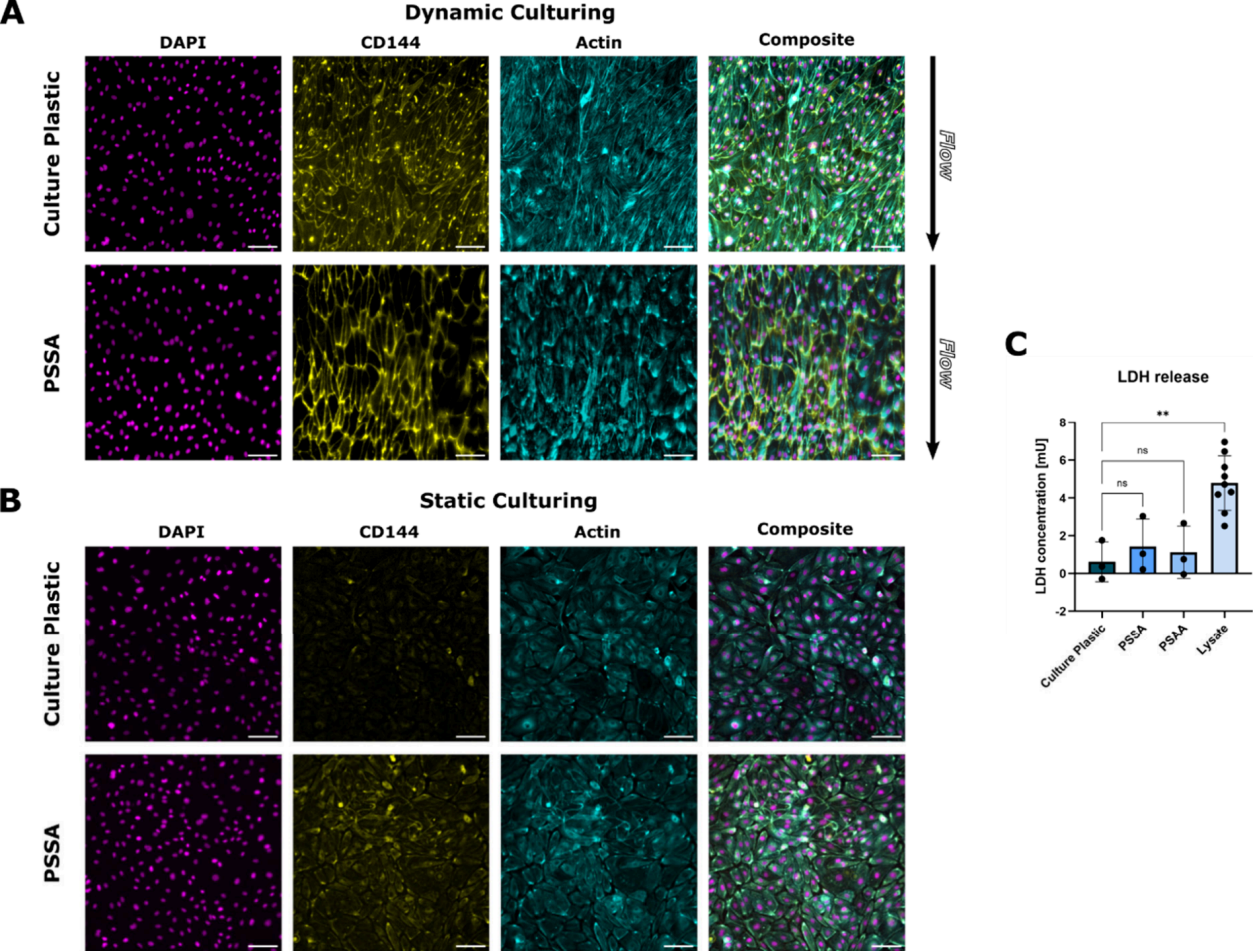
Primary endothelial cells under flow. (A) Representative immunofluorescence stainings of hPMECs cultured for 5 days under 2.3 dyn/cm^2^ shear flow on regular culture plastic and PSSA. Nuclei (magenta; DAPI), endothelial adherens junctions (yellow; VE-cadherin, aka CD144), F-actin (cyan; phalloidin stain). (B) Representative immunofluorescence stainings of hPMECs cultured for 5 days in the chip without shear flow. (C) Lactate dehydrogenase release in culture supernatants. The data points for each condition represent 3 independent culture replicates from 1 donor. The top of the column represents the mean. The whiskers represent the standard deviation. Significance was assessed using one-way ANOVA followed by Tukey’s multiple comparisons test; ** = *p* < 0.01. Scale bars: 100 μm.

To explore the use of PSSA for forming 3D tissue geometries, we seeded hSAECs on rectangular sheets that were transformed into “airway tubes” upon differentiation. We established two airway tube variants: a small airway tube with a 1.5 mm diameter and a large airway tube with a 2.5 mm diameter, both proportioned according to the length-to-diameter ratio of 3 established by Weibel’s morphometric model of the human respiratory tree.^11^ We tested the robustness of the manual folding process and found that only three out of 11 cell-free tubes were misfolded (Table S1, Figure S3) and only one out of eight cell-containing tubes were misfolded (Table S3). We also tested the stability of folded structures over time and found that all cell-free tubes retained their shape (Table S2) and three cell-containing tubes unfolded after 7 days in culture (Table S4). The airway tubes were initially cultured as 2D sheets, differentiated as such for 17 days under submerged conditions, and subsequently rolled into cylindrical structures containing the cells inside. Next, the ciliary beat was recorded in real-time using live staining with tomato lectin, demonstrating tissue integrity upon folding (Figure 4A, SI, videos 1, 2, and 3). This method could be potentially used to generate perfusable airway tube models for drug delivery and airway disease studies.^12^ However, as silicone can absorb and adsorb small molecules, predicting drug response may be challenging.^13^ Thus, further evaluation of the suitability is required.

**Figure 4:**
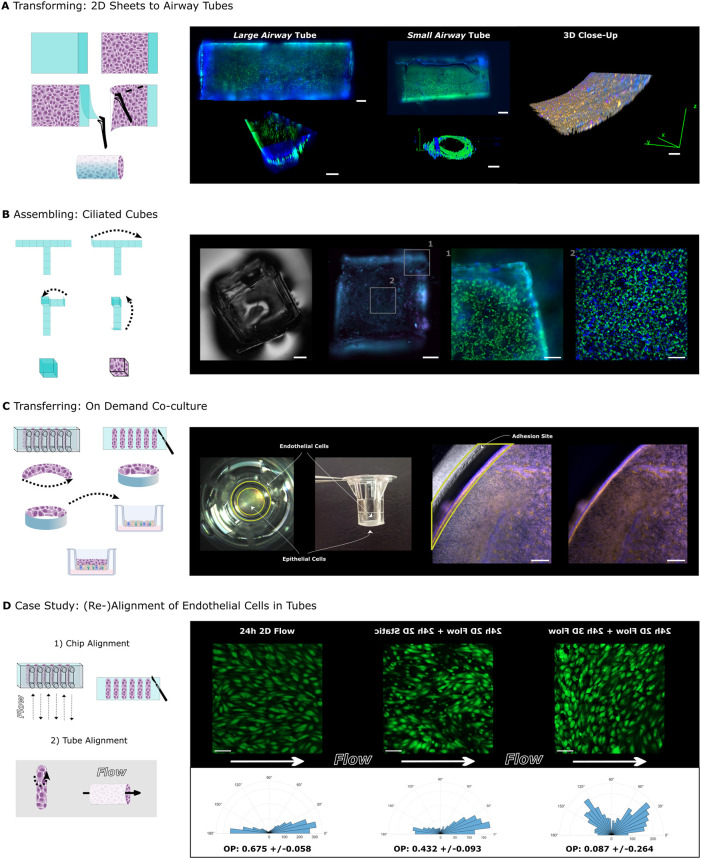
Culture transformation, assembly, and transfer. (A) Left: Schematic of the 2D culture transformation to a 3D airway tube. Right: Live stainings and associated 3D renderings of a large and small airway tube. Cilia (green; *Lycopersicon esculentum* (tomato) lectin), nuclei (blue; Hoechst 33342). 3D Immunofluorescence close-up of fixed tube culture. Cilia (magenta; α-tubulin), nuclei (blue; DAPI), F-actin (orange; phalloidin stain)., (B) Left: Schematic of the assembly of a cube culture. Right: Phase-contrast image of assembled cube without cells. Immunofluorescence staining of fixed cube culture. Cilia (magenta; α-tubulin), F-actin (cyan; phalloidin stain). Close-ups from live stained cube culture. Cilia (green; *Lycopersicon esculentum* (tomato) lectin), nuclei (blue; Hoechst 33342). (C) Left: Schematic of the transfer of shear-aligned endothelial cells to airway epithelial culture in inserts. Right: Photographs show the respective position of the shear-aligned endothelial cells on PSSA from side and top in insert. Immunofluorescence staining of endothelial cells and epithelial cells in insert overlaid with phase-contrast recording (left) to show positioning of PSSA and without phase-contrast overlay (right). Nuclei (blue; DAPI), F-actin (orange; phalloidin stain). Yellow ring marks the position of the adhesive in the inset. (D) Left: Schematic of case study, in which endothelial cells are prealigned in chips and then rolled into tubes for 3D perfusion. Right: Images of GFP-HUVECs (green) after alignment in the chip, 24 h static postalignment, 24 h after perfusion as tube culture in the direction of the initially imposed flow direction. Direction of flow indicated by white arrows. Below are polar histograms of cellular orientation angles relative to flow (flowing from 180° to 0°) from pooled replicates (2–3 replicates, 3 FOVs each) and corresponding orientational order parameters (mean ± standard deviation), 3 FOVs each) and corresponding orientational order parameters (mean ± standard deviation). Scale bars: (A) Tube culture scale bars: large and small airway top maximum projection images: 500 μm; large airway 3D rendering, 1 mm; small airway 3D cross-section, 100 μm; 3D close-up, 50 μm. (B) Cube culture scale bars; entire cubes, 500 μm; close-ups, 200 μm. (C) Insert scale bars: 200 μm. (D) GFP-HUVECs scale bars: 100 μm.

Next, we explored the potential of this technique for constructing diverse 3D geometries before tissue growth. To demonstrate this, we first assembled a millimeter-scale cube using PSSA tape cut into a cube net and then seeded and differentiated hSAECs on its surfaces. The resulting epithelium developed functional motile cilia on all sides (Figure 4B).

Further, to demonstrate the adaptability of this technique for integrating cells with different culture requirements, we prealigned hPMECs under flow for 5 days before transferring them into differentiated hSAEC insert cultures (Figure 4C). This approach highlights the capability of our system to facilitate on-demand coculturing of cells with different preconditioning, differentiation, and microenvironmental needs in a straightforward manner.

Finally, to demonstrate the value of our methodology for addressing fundamental scientific questions, we designed a capstone case study where we tested the dynamic response of endothelial cells to changes in fluid shear stress and substrate geometry (Figure 4D). We first aligned endothelial cells (GFP-HUVECs) grown on PSSA in a microfluidic chip using 2D shear flow (Figure 3). After 24 h, we stopped the flow in some cultures (2D static), whereas other cultures were removed from the chip, rolled up into a 3D tube, and perfused at same shear rate and direction as before (3D flow). As expected, in 2D static conditions, the cellular alignment was slightly reduced after 24 h, as reflected in a reduction of the orientational order parameter (OOP) from 0.68 ± 0.06 (mean ± STD) in the 2D flow compared to 0.43 ± 0.09 in 2D static. An OOP of 1 indicates parallel alignment with the flow direction, −1 indicates perpendicular alignment, and 0 reflects random orientation. Surprisingly, HUVECs in 3D flow conditions, despite perfused with the same shear stress direction and magnitude as on-chip, nonetheless reoriented. The histogram of orientation angles reveals the presence of two peaks, indicating that the cells oriented along two slanted angles relative to the flow. This results in an OOP near zero (0.08 ± 0.26) due to the inability of this measure to distinguish between random and bimodal angle distributions. This realignment might be a response to compressive forces due to conversion from a 2D sheet to a 3D tube.^14^ Taken together, our case study highlights how the ability to release, transfer, and fold cells cultured on PSSA enables the study of fundamental mechanisms.

## Discussion

Our study establishes pressure-sensitive silicone-based adhesives (PSSA) as a flexible substrate for primary cell culture and 3D tissue engineering. The adhesive’s unique properties (optical transparency, low autofluorescence, biocompatibility, gas permeability, resistance to common solvents, PCR compatibility, autoclavability, and mechanical flexibility) facilitate common readouts and preserve tissue integrity. We showed that our flexible cell substrates allow for transferring, reassembling, and 3D-folding of mature 2D tissues with minimal fabrication efforts. Future applications could include biohybrid systems, such as soft robotics,^15,16^ and sensor integration using glued-on interdigitated electrodes^17^ (IDEs) for resistance and capacitance measurements or oxygen and pH sensors^18^ for live monitoring.

Polymer-based thin films have been used previously as flexible epithelial cell culture substrates, contributing to bioinks, removable drug delivery structures, advanced wound dressings,^19^ as well as contractile shapes^20^ and microswimmers,^21^ often using temperature-sensitive coatings^22^ or silicone-based soft skin adhesives (SSAs)^23^ to enable transfer and release. The most sophisticated systems employ computerized pneumatic actuation to achieve transformation between 2D and 3D geometries.^24^ In latest developments, 4D bioprinting combines the shape-shifting properties of materials with 3D printing to create self-folding tubes^25^ and origami-inspired designs.^26^ While these approaches are highly innovative, they require specialized equipment and expertise. Our method provides an affordable and low-tech alternative.

To demonstrate the advantages of our technique, we presented three conceptual applications, followed by a capstone experimental study. First, we engineered airway tubes of various diameters that could enable the assembly of complex branched respiratory tree models. Second, we assembled a millimeter-sized cube featuring a differentiated airway epithelium with active ciliary beating, which could inspire self-propelling soft robotics applications. Third, we leveraged our method for the on-demand coculture of separately cultured human pulmonary microvascular endothelial cells and differentiated airway epithelial cells, facilitating controlled integration of vascular and epithelial components for interaction studies. Finally, we performed a case study to address a fundamental scientific question with our method: how do shear-aligned endothelial cells respond to dynamic changes in geometry? We found that shear-aligned 2D cultures of endothelial cells dynamically realigned to a slanted angle relative to the flow direction after they were converted to perfused 3D tubes. This setup enabled the assessment of competing mechanical cues within the same cell culture and at different time points, i.e., shear stress and radial compressive forces introduced by rolling the cell sheet into a tube. While we provided a proof-of-concept, our results invite many more questions that could be explored with our methodology by, for example, varying the curvature of the tubes, seeding the tubes directly, or perfusing the endothelial sheets with flow perpendicular to their original alignment.

Our study does have limitations. Further studies are needed to evaluate long-term culture of different cell types on PSSA and its effects on cell behavior and potential absorption of small hydrophobic molecules by silicone. Additionally, different extracellular matrix proteins and hydrogels should be tested to enable tissue-specific coculture applications, such as the introduction of pericytes to the endothelial cell model to increase physiological relevance.^27–29^ Using the cutting plotter and manual folding assures affordability and accessibility of the method but limits resolution to >250 μm and requires precise handling, often under sterile conditions, which inherently limits throughput. The PSSA cannot be repositioned after contact with itself, though slight adjustments are possible if the gluing surface is wet. While Young’s Modulus for similar PSSAs in the range of ∼0.6–1.5 MPa^30^ and shear force-to-failure in the range of 0.5–0.8 MPa^6^ have been reported, a more systematic in situ characterization of the mechanical properties of the PSSA in the folded constructs will be an important direction for future studies. The reported elastic modulus of arteries and veins measured via indentation lies in the range of 6.5–560 kPa^31^ matching the lower end of the reported range for PSSAs, however, most soft tissues are more compliant. Soft tissues typically display stiffnesses in the order of 0.1 kPa to hundreds of kPa.^32^ Therefore, incorporating hydrogels or other ECM components to match the stiffness of the tissue of interest, or modifying the composition of the PSSA to create low modulus adhesives (one study reported a range of 2–499 kPa^33^) is essential for achieving physiologically relevant stiffnesses that in turn affect tissue function.^34^ Further, the adhesive film is not porous, which would be needed for studies involving direct cell–cell interactions and medium permeability, such as FITC dextran permeability assays or classical transepithelial electrical resistance (TEER) measurements with voltage-ohm meters and chopstick electrodes for barrier assessments. Porosity could be introduced in the future by layering foamed silicone adhesive on a track-etched membrane carrier. Alternatively, incorporating IDEs as suggested above, would not require porosity while enabling impedance spectroscopy, which not only measures the TEER-equivalent paracellular resistance (tight junction integrity) at low frequencies but also transcellular/capacitive properties (cell morphology, adhesion, membrane capacitance),^35^ providing rich real-time information on cell health, dynamics, and microenvironment.

## Conclusion

Our study establishes silicone adhesives as a viable substrate for primary airway epithelial and endothelial cell culture and flexible 3D tissue engineering, further expanding the scope of adhesive-based substrates beyond traditional material-bonding applications. This adhesive-based system overcomes limitations in a conventional cell culture by combining substrate flexibility and multicellular compatibility. Due to its simplicity and affordability, this method is easily transferable between laboratories and adaptable to diverse cell culture requirements. Future investigations could enhance its potential for advanced *in vitro* modeling, drug screening, biohybrid actuator development, and regenerative medicine by including sensor integration and fabrication scaling for high-throughput biomedical applications, advancing preclinical models as well as biohybrid robotics.

## Supplementary Material









## Notes

Additional experimental details, materials and methods, supporting figures of cell type composition of airway epithelial cultures and primary cell proliferation on PSSA (PDF)
Video 1: Cilia motion video from a top view in tube culture (AVI) (AVI)
Video 2: Cilia motion video from a side view in tube culture (AVI) (AVI)
Video 3: Cilia motion video from a close-up in tube culture (AVI) (AVI)
