# Supplementary material for: Release, Transfer, Fold: Using a Silicone Adhesive for On-Demand 3D Tissue Engineering
Source: ACS Biomater Sci Eng. 2025 Oct 11;11(11):6506–14. doi: 10.1021/acsbiomaterials.5c01130 (PMC12606553; doi:10.1021/acsbiomaterials.5c01130)
Supplement: Supplementary file 1 [file ab5c01130_si_001.pdf]

Release, Transfer, Fold: Using a Silicone Adhesive for On-Demand 3D Tissue Engineering

Doris Roth, Benedetta Zampa, Romina Augustin, Daara Payandehjoo, Giancarlo Porcella, Ayşe Tuğçe Şahin, Anne M. van der Does, Janna C. Nawroth*

**Corresponding author. E-mail:* janna.nawroth@helmholtz-munich.de

1. Experimental Details S2
   Rapid Prototyping and Fabrication S2
   Cell Culture S3
   IF Staining and Imaging S4
   Cell Type Composition Analysis S4
   Ciliary Beating Life-Imaging S4
   Ciliary Beat Frequency Imaging and Analysis S4
   LDH Assay S4
   Cell Proliferation S5
   Orientational Order S5
   Statistics S5
2. Supplementary Figures S6
3. Supplementary Tables S8
4. References S9

1. Experimental Details

Rapid Prototyping and Fabrication

In this study, two types of biomedical pressure-sensitive adhesives were used to diversify cell culture formats and conditions: a clear silicone-based adhesive (SR-29) lining both sides of a polypropylene film (ARcare 94119, Adhesive Research; PSSA) with a total thickness of 142 µm, and an acrylic-based adhesive (AS-110) lining both sides of a polyester film (ARcare 90445Q, Adhesive Research; PSAA) with a total thickness of 81 µm.

All adhesive shapes were designed using Inkscape and exported to CutStudio (Roland) or directly designed in CutStudio for simpler geometries. Shapes were cut using a CAMM-1 Servo GX-25 (Roland) plotter cutter equipped with a ZEC-U5032 (Roland) blade, set to a cutting force of 100 g and a speed of 5 mm/s. Following cutting, the adhesive shapes were sterilized with ethanol and treated with UV light to ensure sterility for cell culture applications.

Control Cultures. To compare on-adhesive cell cultures with conventional insert cultures, adhesive sheets were cut into circles matching the culture area of standard inserts (0.32 cm²). The release layers of the adhesive circles were removed. Each circle was then affixed to the bottom of a 96-well plate for use in control experiments.

Preparation of Adhesive Rectangles for 2D Cultures Designed to Form Tubular Structures. Rectangles were prepared for 2D culture systems with the ability to roll into tubes. Two tube diameters were selected: 2.5 mm to model large airways (>2 mm) and 1.5 mm to model small airways (<2 mm). Using the approximate length-to-diameter ratio established by Weibel et al.[1], and accounting for an additional 1 mm width for adhesive overlap during tube formation, rectangles measuring 7.5 x 8.85 mm and 4.5 x 5.7 mm were cut. A longitudinal slit was made 1 mm from the left edge of each rectangle, cutting only the top release layer. The release layer over the culture area (7.5 x 7.85 mm and 4.5 x 4.7 mm, respectively) was removed. The rectangles were then affixed to the bottom of an 8-well slide (µ-Slide 8 Well high, ibidi) using a circular cut of the adhesive. This method secured the rectangles to prevent floating while retaining the bottom release layer, enabling subsequent rolling into tubes.

Preparation of Adhesive Cube Net for 2D Cultures Designed to Form Cubes. A cube net was designed that glues to itself and cut with small incisions at 2 mm intervals to facilitate assembly, resulting in a 2 mm x 2 mm cube. After cutting, the net was carefully bent along the incisions to create creases for precise folding. Under sterile conditions, the release layers were removed, and the net was folded into a cube using tweezers. Once assembled, the cubes were immediately immersed in coating solution to prevent them from adhering to any surfaces

Preparation of Adhesive Rectangles for 2D Flow-Aligned Endothelial Cultures for Transfer or Tubular Structure Formation. For endothelial perfusion cultures, an adhesive rectangle was cut to match the dimensions of a bottomless single- or six-channel slide (25.5 x 75.5 mm², µ-Slide I/IV, ibidi). The release layer on one side of the adhesive was removed, and the adhesive was affixed to the autoclaved channel slide using manual pressure. The second release layer remained intact to facilitate later handling and potential transfer.

Folding Success and Stability. To evaluate the success rate of the folding process and whether the shape is maintained over time, images of the tubes with and without hPMECs were taken immediately

after folding and again after one week in culture for cell-containing tubes. For each timepoint three images were taken: one from above, and two showing the cross-section of the tube from each side. From these images, we obtained three measurements: length and two measures of tube diameter (diameter 1: width of each tube end seen from side; diameter 2: cross-sectional width of each tube end from top-view). Having calculated the mean diameter from both diameter measures, we assessed the average diameter-to-length ratio of the tubes, considering an error margin of 10% to be acceptable and labelling all tubes with a greater discrepancy as misfolded. See supporting table S1, S2, S3 and S4.

Cell Culture

Human primary small airway epithelial cells (hSAECs) and human primary pulmonary microvascular endothelial cells (hPMECs) were obtained from Lifeline Cell Technologies (USA). hSAECs (passage 1) and hPMECs (passages 2-3) were expanded in tissue culture-treated T75 flasks (Corning) using complete bronchial epithelial cell medium (BEpiCM, ScienCell) or complete endothelial cell growth medium-MV2 (ECGM-MV2, PromoCell) until ~90% confluence was reached. GFP-labeled human umbilical vein endothelial cells (GFP-HUVECs) were gifted from the Matthias Meier Lab at Helmholtz Munich. They were expanded on 5µg/mL fibronectin (Corning) coated T25 flasks in ECGM-MV2 Medium (Promocell).

Epithelial Cell Culture Protocol. 24-well PET membrane culture inserts (pore size 0.4 µm, Transwell Corning) for control cultures and adhesive shapes were coated overnight with 300 µg/mL human collagen type IV (Sigma-Aldrich). The following day, the collagen coating was removed, and the culture surfaces were washed with PBS. Expanded hSAECs were harvested by incubating the cells in the T75 flask with Accutase (Thermo Scientific) for 20min and subsequent pelleting of the cell suspension for 7min at 210g. hSAECs were then seeded at 250,000 cells/cm² density in BEpiCM medium. Following overnight attachment, the medium was switched to PneumaCult-ALI™ medium (STEMCELL Technologies) supplemented with 10 µM DAPT (Thermo Scientific Chemicals) for differentiation under liquid conditions, following established protocols.[2] For air-liquid interface (ALI) control cultures, the medium was replaced with PneumaCult-ALI™ medium, and cultures were air-lifted to promote differentiation. For cube cultures, the cubes were submerged in a cell suspension containing 1,000,000 cells/mL BEpiCM and placed on a rocker at lowest speed for one hour. The next day after static overnight culturing, the medium was replaced with PneumaCult-ALI™ medium supplemented with 10 µM DAPT. Cubes on which all faces were covered with cells were cultured for 17 days.

Endothelial Cell Culture Protocol. For endothelial cultures, channel slides with a tissue culture-treated polymer coverslip bottom (µ-Slide I/IV, ibidi) or assembled channel slides with an adhesive bottom were coated overnight with 50 µg/mL human fibronectin (Corning). The next day, the slides were washed with PBS. Expanded hPMECs were seeded into the channels at a density of 300,000 cells/cm² and allowed to attach for 1 hour. After attachment, the cells were cultured under physiological low shear stress (2.3 dynes/cm²) for 5 days using the ibidi Pump System. Static controls were maintained in parallel, with medium changes every other day.

Case Study: GFP-HUVECS were initially cultured in microfluidic channels, as described above, at 10 dynes/cm² for 24 hours to achieve alignment. Next, the PSSA was peeled from the channel slides and individual endothelial cultures were excised using a scalpel. The excised cultures were glued to a second PSSA, which was then rolled to form a tube. The resulting tubes were either connected to the ibidi pump system and sealed with parafilm to prevent possible leakage at tube and tubing interface (Figure S4) or kept static. The perfused *cultures w*ere exposed to 10 dynes/cm² for 24 hours. Control cultures were left in 2D after the initial 24h of perfusion on the chip to assess loss of alignment.

IF Staining and Imaging

After differentiation or flow alignment, primary human cultures were fixed with 4% PFA for 30 minutes at room temperature, washed with PBS, and stored in PBS at 4°C until staining. Samples were blocked and permeabilized using 0.25% Triton-X 100 in PBS with 3% BSA for 1 hour at room temperature, followed by overnight incubation at 4°C with primary antibodies against MUC5AC (abcam, 1:150 dilution) and Uteroglobin/SCGB1A1 (Proteintech, 1:100 dilution) for hSAECs and CD144 (eBioscience, 1:200 dilution) for hPMECs. After rinsing three times for five minutes with PBS, secondary antibodies and subsequently directly conjugated anti-acetylated-α-tubulin (Santa Cruz, 1:200) for hSAECS and phalloidin (F-Actin, Thermo Fisher Scientific, 1:1000) for both hSAECs and hPMECs as well as DAPI, were applied in Triton-X/BSA buffer for 1 hour at 37°C, followed by PBS washes. 2D samples were mounted on glass slides with ProLong™ Glass Antifade Mountant (Thermo Scientific), while 3D samples were placed in imaging dishes with SlowFade™ Glass Antifade Mountant (Thermo Scientific) or PBS. Imaging was conducted using a Zeiss Axio Observer fluorescence microscope and a 40× oil objective, capturing 6 fields of view (FOVs) with a size of 2048 × 2048 pixels and 6.2μm/pix resolution per stained sample. For the 3D close-up for the airway tube imaging was conducted using an Andor BC43 CF benchtop confocal microscope and a 40x oil objective.

Cell Type Composition Analysis

To quantify the composition of cell types from immunofluorescence images with three channels, namely MUC5AC, SCGB1A1, and acetylated α-tubulin (ATUB)[3], we made binary masks of each signal per FOV and computed the area covered by the signal using Fiji ImageJ[4].

Ciliary Beating Life-Imaging

Cilia were live-stained with fluorescent-dye conjugated tomato lectin diluted in PBS (Lycopersicon Esculentum (Tomato) Lectin DyLight488, Invitrogen, dilution 1:40)[3] and Hoechst 33342 (Thermo Scientific, dilution 1:1000) by incubating the sample in the solution for 20 minutes. After rinsing with medium, ciliary beat kinematics were recorded at 30 fps using epifluorescence imaging on an inverted Zeiss Axio Observer with a 40x (NA 0.8) phase contrast objective, a temperature-controlled chamber set to 37°C, and an Orca Flash 4.0 camera (Hamamatsu).

Ciliary Beat Frequency Imaging and Analysis

Ciliary beat frequency (CBF) was recorded using a Zeiss Axio Observer microscope with oblique phase contrast, an Orca Flash 4.0 camera, and a temperature-controlled chamber set to 37°C. High-speed movies (ca. 140 frames per second for at least 1.5 s) were captured with a 40× long-distance phase contrast objective at a resolution of 0.3 μm/pixel in a 512 × 512-pixel frame. For each culture, 5-10 FOVs were recorded. Cilia beat frequencies were measured using the FreQ plugin[5] in Fiji ImageJ.

LDH Assay

LDH was measured using the LDH-Glo Cytotoxicity Assay (Promega) according to the instructions of the manufacturer. Measurements were carried out in technical triplicates. Maximum LDH release was determined by lysing the cells in 10% Triton X-100 for 20min. Since culture vessel dimensions and culture areas varied, measurements were normalized to both supernatant volume and culture area.

The data are expressed in milliunits following the standard curve method in the linear range of the assay.

Cell Proliferation

To evaluate the effect of PSSA on cell proliferation, 6 mm diameter circles of the PSSA were sterilized, cut, and affixed to a 96-well plate. The plate was subsequently coated as described above. SAECs and hPMECs were seeded at densities of 65,000 cells/cm² and 3,500 cells/cm², respectively. After a 2-hour incubation period to allow cell attachment, and again at 24 and 48 hours post-attachment, the cultures were stained with Hoechst 33342 (as described in *Ciliary beating life-imaging*) for live-cell imaging. Nuclei were quantified using particle analysis in Fiji ImageJ. Each condition was analyzed in three technical replicates, with three fields of view (665.6 × 665.6 μm) captured per replicate. Cell densities (cells/mm²) were calculated and normalized to the mean baseline value at 0 hours to determine the fold change in cell count.

Orientational Order

Fluorescence images of GFP-HUVECs were acquired in an incubated chamber at 37°C using a 20x Objective and Zeiss Axio Observer. The tube cultures were fixed in 4%PFA for 20min and either GFP was imaged directly, or in samples where the GFP signal was not present anymore, the samples were additionally stained with phalloidin (F-actin, Thermo Fisher Scientific, 1:1000) for 60min at RT. Cell orientation relative to the imposed flow direction was quantified in ImageJ/Fiji.[4] For each image, endothelial cells were segmented using the ImageJ plugin "Mask Of Nearby Points" and the Feret's angle was measured of each cell relative to the flow axis.The orientational order parameter (OOP) was calculated for each replicate according to $OOP=\langle \cos(2\theta) \rangle$, where θ is the angle between the long axis of the cell and the flow direction, and $\langle \cdot \rangle$ denotes the average over all cells analyzed. An order parameter of 1 corresponds to perfect alignment parallel to flow, −1 corresponds to perpendicular alignment, and 0 corresponds to random orientation. Order parameters were computed separately for each technical replicate. Polar histograms were generated using a MATLAB script and all measured angles of replicates were pooled for this purpose.

Statistics

Statistical analysis was performed using GraphPad Prism (version 9, GraphPad Software Inc). A p-value of less than 0.05 was considered statistically significant. Details on statistical significance and the number of donors and samples are provided in the figures and figure legends.

2. Supplementary Figures

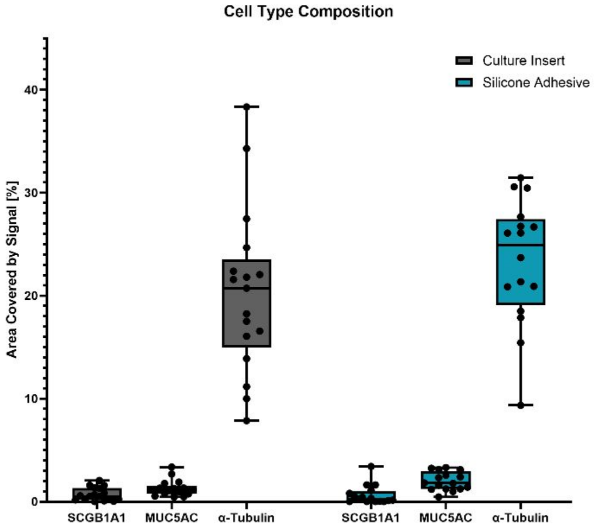

Figure S1 Cell type composition of submerged airway culture on insert and on pressure-sensitive silicone adhesive (PSSA).

Quantification of area covered by immunofluorescent signals of cell type markers SCGB1A1 (club cells), MUC5ac (goblet cells) and α-tubulin (ciliated cells) in submerged and silicone adhesive hSAEC cultures at day 17 of differentiation in a FOV. Data points represent pooled data from 3 culture replicates and a minimum of 4-6 FOVs per culture. Thick horizontal lines represent the median, bottom and top edges of the boxes represent the 25th and 75th percentiles, and whiskers indicate the minimum and maximum.

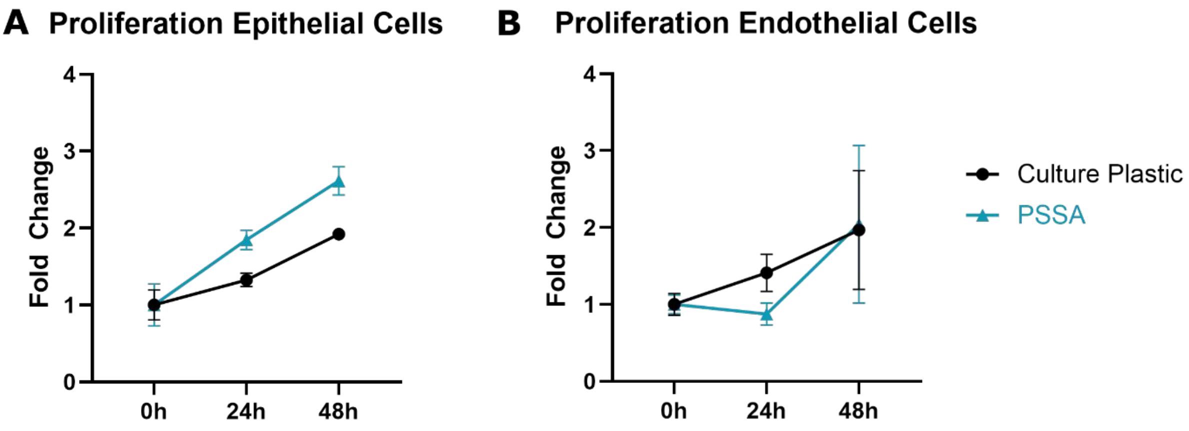

Figure S2 Proliferation of primary airway epithelial and pulmonary microvasculature endothelial cells on culture plastic and PSSA.

Quantification of fold change of cell count from baseline when the cells have attached (=0 hours), 24 hours and 48 hours thereafter. Data points represent the mean fold change to the 0-hour baseline mean of pooled cell counts per mm² from 3 technical replicates and 3 FOVs per replicate. The whiskers indicate the standard deviation.

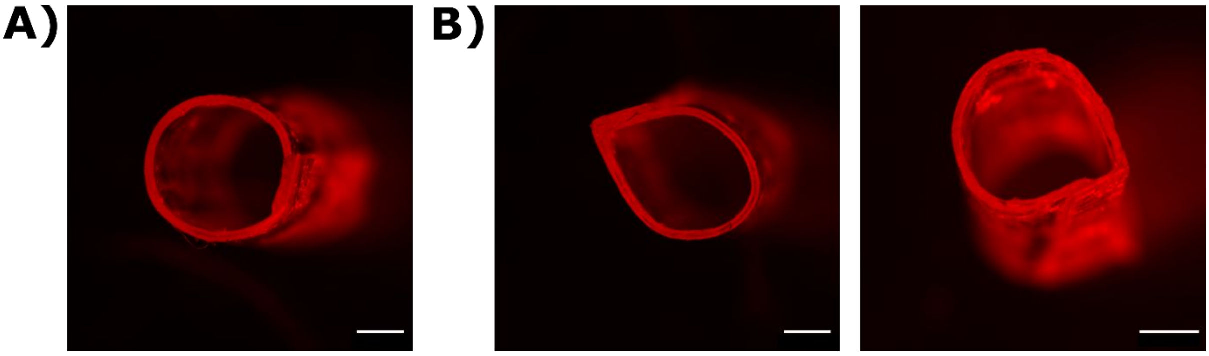

Figure S3 Cross sections of tubes after folding.

(A) Image of a cross section of a correctly folded tube. (B) Image of cross sections of misfolded tubes.Scale bar: 1 mm

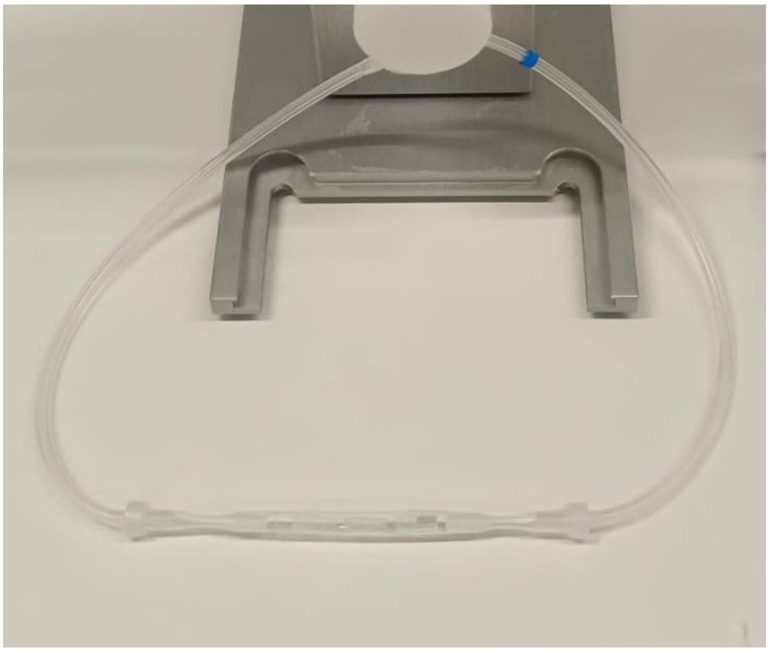

Figure S4 Perfusion of tube culture.
The endothelial cultures are affixed to an additional PSSA that is glued to cut p10 pipette tips (Eppendorf) and rolled to form a tube. The circuit is then sealed with parafilm at the connection points.

3. Supplementary Tables

Tube Number	D1 [mm] Opening 1	D1 [mm] Opening 2	D2 [mm] Opening 1	D2 [mm] Opening 2	Mean Diameter	Length [mm]	L/D	% Deviation from Expected L/D	Allow ±10% deviation
Expected	2.5	2.5	2.5	2.5	2.5	7.5	3	0	Pass
1	2.73	2.23	2.45	2.41	2.45	7.39	3.01	0.003	Pass
2	3.21	2.81	2.88	2.92	2.95	7.40	2.51	0.165	Misfolded
3	2.56	2.53	2.51	2.47	2.52	7.43	2.95	0.017	Pass
4	2.94	2.17	2.69	2.64	2.61	7.53	2.88	0.039	Pass
5	2.16	2.73	2.69	2.78	2.59	7.42	2.87	0.044	Pass
6	2.66	2.44	2.50	2.59	2.55	7.26	2.85	0.051	Pass
7	4.26	2.59	2.71	2.74	3.07	7.48	2.43	0.189	Misfolded
8	2.82	2.64	2.93	2.86	2.81	7.37	2.62	0.126	Misfolded
9	2.54	2.27	2.72	2.63	2.54	7.43	2.93	0.025	Pass
10	2.51	2.36	2.65	2.69	2.55	7.44	2.91	0.029	Pass
11	2.69	2.63	2.55	2.54	2.60	7.55	2.90	0.032	Pass

Table S1 Geometrical characterization of folded tubes without cells.
For each tube, the opening diameters as seen from both sides (D1), the cross-section from both ends (D2) as seen from the top and the length (L) of the tube were measured after folding. The length-to-diameter ratio (L/D) was measured as the ratio between L and the mean of the four diameter values. L/D was then compared with the expected reference value to assess folding quality. A deviation within ±10% of the expected L/D was considered acceptable, whereas larger deviations were classified as misfolded.

Tube number	D1 [mm] Opening 1	D1 [mm] Opening 2	D2 [mm] Opening 1	D2 [mm] Opening 2	Mean Diameter	Length [mm]	L/D	% Deviation from Expected L/D	Allow ±10% deviation
Expected	2.5	2.5	2.5	2.5	2.5	7.5	3	0	Pass
1	2.42	2.62	2.48	2.78	2.57	7.51	2.91	0.028	Pass
2	2.43	2.54	2.64	2.53	2.53	7.61	3.00	0.001	Pass
3	2.60	2.26	2.50	2.65	2.50	7.61	3.04	0.014	Pass
4	2.58	2.58	2.67	2.59	2.60	7.59	2.92	0.028	Pass
5	2.60	2.44	2.65	2.55	2.56	7.59	2.96	0.012	Pass
6	2.48	2.45	2.73	2.73	2.60	7.58	2.92	0.027	Pass

Table S2 Geometrical characterization of folded tubes without cells after 5 days submerged in water.

Tube number	D1 [mm] Opening 1	D1 [mm] Opening 2	D2 [mm] Opening 1	D2 Opening 2	Mean Diameter	Length [mm]	L/D	% Deviation from Expected L/D	Allow ±10% deviation
Expected	2.5	2.5	2.5	2.5	2.5	7.5	3	0	Pass
1	2.50	3.23	2.66	2.57	2.74	7.23	2.64	0.120	Misfolded
2	2.57	2.50	2.71	2.62	2.60	7.15	2.75	0.084	Pass
3	2.60	2.69	2.59	2.72	2.65	7.32	2.76	0.079	Pass
4	2.56	2.52	2.56	2.63	2.57	7.28	2.83	0.056	Pass
5	2.71	2.42	2.71	2.63	2.62	7.49	2.86	0.047	Pass
6	2.51	2.78	2.72	2.52	2.63	7.35	2.79	0.070	Pass
7	2.64	2.80	2.53	2.51	2.62	7.52	2.87	0.043	Pass
8	2.41	2.77	2.69	2.71	2.64	7.49	2.83	0.057	Pass

Table S3 Geometrical characterization of folded tubes seeded with hPMECs.

Tube number	D1 [mm] Opening 1	D1 [mm] Opening 2	D2 [mm] Opening 1	D2 [mm] Opening 2	Mean Diameter	Length [mm]	L/D	% Deviation from Expected L/D	Allow ±10% deviation
Expected	2.5	2.5	2.5	2.5	2.5	7.5	3	0	Pass
1	-	-	-	-	-	-	-	-	Unfolded
2	2.21	2.40	3.37	2.85	2.71	7.42	2.74	0.087	Pass
3	2.32	2.46	2.42	2.81	2.50	7.29	2.91	0.029	Pass
4	2.52	2.74	2.41	2.43	2.53	7.44	2.940	0.020	Pass
5	-	-	-	-	-	-	-	-	Unfolded
6	-	-	-	-	-	-	-	-	Unfolded
7	2.31	2.68	2.51	2.81	2.58	7.39	2.87	0.045	Pass
8	2.69	2.75	2.65	2.53	2.66	7.55	2.84	0.053	Pass

Table S4 Geometric characterization of folded tubes seeded with hPMECs 7 days after folding.

4. References

(1) Weibel, E. R. The Structural Basis of Lung Function. In *Respiratory Physiology*; West, J. B., Ed.; Springer New York: New York, NY, 1996; pp 3–46, DOI: 10.1007/978-1-4614-7520-0_1.

(2) Gerovac, B. J.; Valencia, M.; Baumlin, N.; Salathe, M.; Conner, G. E.; Fregien, N. L. Submersion and Hypoxia Inhibit Ciliated Cell Differentiation in a Notch-Dependent Manner. *Am J Respir Cell Mol Biol* **2014**, *51* (4), 516–525, DOI: 10.1165/rcmb.2013-0237OC.

(3) Roth, D.; Şahin, A. T.; Ling, F.; Tepho, N.; Senger, C. N.; Quiroz, E. J.; Calvert, B. A.; Van Der Does, A. M.; Güney, T. G.; Glasl, S.; Van Schadewijk, A.; Von Schledorn, L.; Olmer, R.; Kanso, E.; Nawroth, J. C.; Ryan, A. L. Structure and Function Relationships of Mucociliary Clearance in Human and Rat Airways. *Nat Commun* **2025**, *16* (1), 2446, DOI: 10.1038/s41467-025-57667-z.

(4) Schindelin, J.; Arganda-Carreras, I.; Frise, E.; Kaynig, V.; Longair, M.; Pietzsch, T.; Preibisch, S.; Rueden, C.; Saalfeld, S.; Schmid, B.; Tinevez, J.-Y.; White, D. J.; Hartenstein, V.; Eliceiri, K.; Tomancak, P.; Cardona, A. Fiji: An Open-Source Platform for

Biological-Image Analysis. *Nat. Methods* **2012**, *9* (7), 676–682, DOI: 10.1038/nmeth.2019.
(5) Jeong, I.; Hansen, J. N.; Wachten, D.; Jurisch-Yaksi, N. Measurement of Ciliary Beating and Fluid Flow in the Zebrafish Adult Telencephalon. *STAR Protocols* **2022**, *3* (3), 101542, DOI: 10.1016/j.xpro.2022.101542.
